# Effectiveness of a tailored implementation programme to improve recognition, diagnosis and treatment of anxiety and depression in general practice: a cluster randomised controlled trial

**DOI:** 10.1186/s13012-015-0210-8

**Published:** 2015-03-12

**Authors:** Henny Sinnema, Maria Cristina Majo, Daniëlle Volker, Adriaan Hoogendoorn, Berend Terluin, Michel Wensing, Anton van Balkom

**Affiliations:** Netherlands Institute of Mental Health and Addiction, Trimbos Institute, 3500 AS Utrecht, The Netherlands; Department of Psychiatry, VU University Medical Centre and GGZinGeest, 1081 HL Amsterdam, The Netherlands; Department of General Practice and Elderly Care Medicine, EMGO Institute for Health and Care Research, 1000 SN Amsterdam, The Netherlands; Radboud University Medical Centre, Scientific Institute for Quality, 6500 HB Nijmegen, The Netherlands

**Keywords:** Anxiety, Depression, Primary care, Tailored interventions, Implementation science, Cluster randomised controlled trial

## Abstract

**Background:**

Anxiety and depression are not always diagnosed and treated in primary care as has been recommended. A tailored implementation programme, which addresses key barriers for change by targeted interventions, may help to remedy this.

**Methods:**

The effectiveness of an individually tailored implementation programme, additional to standardised training and feedback on the recognition and treatment of patients with anxiety or depression in general practice, was examined in a cluster randomised controlled trial. Participants were 46 general practitioners (GPs) from 23 general practices (12 intervention, 11 control) and 444 patients aged 18 years or older (198 intervention, 246 control) who screened positive on the extended Kessler 10. In the control group, GPs received a 1-day training in guidelines for recognition and stepped treatment for anxiety and depression. Ten months after the training session, GPs received feedback on their performance over the preceding 6 months. In the intervention group, GPs received the same training and feedback as those in the control condition; in addition, they were offered support, tailored to perceived local barriers to change. The support was delivered in the format of peer group supervisions and personalised telephone consultations. Data were based on an audit of patient records and patient surveys at baseline and after 3 and 6 months.

**Results:**

The tailored implementation programme led to recognition of a higher proportion of patients presenting with anxiety and depression (42% versus 31%; odds ratio (OR) = 1.60; 95% CI: 1.01–2.53), more consultations after recognition (IRR = 1.78; 95% CI: 1.14–2.78) and did not lead to more prescription of antidepressants (OR = 1.07; 95% CI: 0.52–2.19) or referral to specialist mental health services (OR = 1.62; 95% CI: 0.72–3.64). Patients in the intervention group reported better accessibility of care (effect size (ES) = 0.4; *p* < 0.05) and provision of information and advice (ES = 0.5; *p* < 0.05).

**Conclusions:**

A tailored implementation programme may enhance the recognition and treatment of patients with anxiety or depression. Further development and evaluation of the programme is warranted to determine its cost-effectiveness.

**Trail registration:**

Dutch Trial Register identifier NTR1912.

**Electronic supplementary material:**

The online version of this article (doi:10.1186/s13012-015-0210-8) contains supplementary material, which is available to authorized users.

## Background

Anxiety and depression are highly prevalent and have a negative impact on everyday functioning, cause great suffering and incur high healthcare costs and costs associated with reduced productivity [1-3]. In the Netherlands, most adults who seek help for anxiety or depression are treated in general practice [4]. Although clinical guidelines are available [5,6], the management of these disorders in general practice is often suboptimal. Under-recognition of anxiety and depression has been reported, although more severe symptoms may be more easily recognised [7,8]. Only half the patients presenting with anxiety and depression receive care which is in accordance with the guidelines for treatment in general practice [9-11]. There is evidence that adhering to treatment guidelines would produce better patient outcomes [12,13], so improving adherence to the guidelines is an important objective.

A variety of factors may negatively affect adherence to guidelines for the treatment of anxiety and depression. There are barriers related to patients, professionals (i.e. the physicians) and organisations. Many patients do not acknowledge that they suffer from anxiety or depressive symptoms, although they may present in general practice with somatic symptoms instead of mental health symptoms [14-16]. Even when a psychiatric diagnosis is made, the patient or general practitioner (GP) may not perceive treatment as necessary [11]. Barriers to GPs adhering to the guidelines may include problems differentiating between ‘normal’ distress and anxiety or depressive disorders. Some GPs find it difficult to discuss the factors relevant to diagnosis with patients [17]. Possible barriers at the organisational level include insufficient collaboration between GPs and mental health professionals, waiting lists for specialised mental health services and limited financial incentives [18,19].

Adherence to guidelines may be improved by interventions which are tailored to prospectively identified local barriers [20]. Only a few studies have assessed the effectiveness of tailored interventions in improving adherence to guidelines for the management of patients with depression in primary care [21,22]. In one study, all participating GPs received an identical intervention, this study found that GPs in the intervention group were more likely to diagnose depression and prescribe antidepressants, but no effect on health outcomes was detected [21]. Another study showed that a one-off tailored intervention based on psychological theories improved some outcomes, more specifically suicide risk assessment improved and patients’ depressive symptoms decreased [22]. These studies notwithstanding, there is insufficient data to determine the most effective and efficient approach to tailoring this type of intervention. Tailoring an intervention may increase or decrease costs, but we were unable to find reports of economic evaluations of such interventions.

Our hypothesis was that adherence to guidelines for anxiety and depression—and in consequence patient outcomes—would be improved by interventions that are tailored to prospectively identified local barriers affecting GPs. The identification of barriers to implementation of guidelines, the development of interventions targeting these barriers and the application and perceived usefulness of the resulting tailored interventions have been described in detail elsewhere [23]. This is the first study to determine the effectiveness of tailored interventions to improve adherence to guidelines for the recognition of anxiety and depression in general practice. We compared training and feedback for GPs with training and feedback supplemented by a tailored intervention. Training and feedback was provided to both groups. Educational meetings and in less degree feedback are common strategies used with the aim of improving professional practice and patient outcomes. However, the effect is most likely to be small and depending on baseline performance and how they are provided [24,25]. The rationale to provide the training was that the GP needs knowledge of the guideline recommendations in order to be able to adhere to these guidelines. Feedback was chosen to give the GP the opportunity to adjust their performance. Besides, the provision of training and feedback would probably motivate GPs to participate in the study.

## Methods

### Study design

This study was a pragmatic, two-arm, general practice-level cluster randomised controlled trial (RCT) [26]. Clusters were general practices (solo practices, group practices or health centres) in the Netherlands. The study was approved by the Medical Ethics Committee of the Institutions for Mental Health (METiGG; Utrecht, The Netherlands) in 2009, number NL28350.097.09.

The researchers were independent of the funder ZonMw.

### Randomisation of clusters

The general practices were randomly assigned to the intervention group or the control group. GPs in both groups received 1 day of training in recognition, diagnosis, stepped treatment and patient education about anxiety and depression after which randomisation was performed by an independent statistician. GPs were not blind to group assignment. Patients were informed about the project, but did not receive information whether their GP had been allocated to the intervention group or to the control group.

### Eligibility criteria for clusters and participants

#### Setting

The study involved 46 GPs in 23 general practices, who were recruited over a 5-month period from September 2009 onwards. Several recruitment strategies were used: a random sample of 500 GPs derived from a national register and all 225 GPs contracted to a particular health insurance company that made additional payments (€ 0.60 cent for each practice-listed patient in 2010 and 2011) received a newsletter containing information about the goals of the study and the accreditation they would receive if they followed the 1-day training on the use of the guidelines. Subsequently, a researcher contacted these practices by telephone to recommend participation. In addition, information about the study was published on the website of the Dutch College of General Practitioners. The inclusion criterion was a willingness to participate in a 1-day training programme.

#### Patient sample

All patients aged 18 years or older attending the participating general practices between September 2010 and June 2011 received an information letter and an invitation to participate and were asked to complete the extended Kessler 10 (EK-10).

Patients who screened positive on the EK-10 were included in the study. The Dutch EK-10 is a validated instrument for screening for anxiety and depressive disorders in primary care [27]. A screening is considered positive if the patient scores at least 20 on the K10 or gives at least one positive response to the five additional questions about anxiety. Exclusion criteria as assessed by the GP were suicidal ideation and behaviour, dementia and other severe cognitive disorders, psychotic disorder, bipolar disorder, dependence on alcohol or drugs, a severe, unstable somatic condition diagnosed by their GP, insufficient knowledge of the Dutch language to enable accurate completion of the questionnaires, having received psychological treatment in the 6 months before the start of the study and having been diagnosed with anxiety or depression by a GP in the 6 months before the start of the study. Patients with a positive screening on the EK-10 who had consented to being contacted were contacted by telephone and given further information about the study. Patients who met the inclusion criteria received an information letter, the baseline questionnaire and a second informed consent form. Inclusion in the study was confirmed when a patient returned the baseline questionnaire and gave informed consent for participation in the study. GPs were not informed which patients had been included. After the baseline assessment, patients might have consulted their GP, but it is also possible that they did not.

### Interventions

#### Control group and intervention group

##### Training

GPs in both groups received the same set of guidelines and, in February 2010, received a 1-day training session on implementation given by experts in the treatment of anxiety and depression in primary care and in the use of clinical guidelines [28-30]. The intervention focused on four important guideline recommendations relating to recognition, diagnosis, treatment and patient education.

##### Feedback

GPs in both groups were asked to complete a consultation registration form for each patient who completed the Four-Dimensional Symptom Questionnaire (4DSQ). The form included questions on 4DSQ score, diagnosis, the treatment indicated and whether the GP had informed the patient about the diagnosis and stepped treatment options. The consultation registration forms were used to provide GPs with feedback about the number of registered 4DSQs, appropriate diagnoses, treatment allocation and patient education. The feedback was an evaluative assessment of whether patients had been appropriately diagnosed, treated and educated and was provided 6 months after the start of the tailored intervention (December 2010).

1. *Recognition of patients with anxiety or depressive disorders*. The 4DSQ may be used to help recognise anxiety and depressive disorders. This self-report instrument can be used to distinguish between stress-related syndromes (termed ‘stress’, ‘burnout’ and ‘nervous breakdown’) and psychiatric disorders (i.e. anxiety and depressive disorders) [31].
2. *Diagnosis of anxiety disorder or a depressive disorder in patients scoring above a certain threshold on the 4DSQ*. An appropriate diagnosis includes an assessment of the severity of the disorder: ‘simple’ or ‘complex’ for anxiety disorder and ‘mild’ or ‘severe’ for depressive disorder.
3. *Treatment should be determined by reference to a stepped care approach* [32-34]. Treatment should be based on the severity of the disorder and should begin with the least intensive treatment that may be expected to prove effective. Patients with a simple or mild disorder should be offered less intensive intervention; more intensive treatment options are appropriate for patients who have failed to respond to low-intensity interventions and for patients with a complex or severe disorder.
4. *Patient education on anxiety and depression*. GPs should provide patients with information about their diagnosis and the stepped treatment options for anxiety and depression.

#### Intervention group only

##### Tailored intervention

Between June 2010 and June 2011, GPs from general practices randomised to the intervention group received, in addition to the training and feedback, interventions that were tailored to prospectively identified local barriers. To provide insight into the perceived barriers to early recognition of anxiety and depression, appropriate diagnosis, appropriate treatment allocation and patient education, a trained interviewer carried out a semi-structured face-to-face interview with all participating GPs. The interview protocol was developed from a review of studies on barriers to compliance with guidelines for anxiety and depression [18,35-37]. Interviews were conducted; at the time, the baseline for the RCT was taken and yielded a list of barriers relevant to each GP. Different barriers were perceived by the GPs to the uptake of guideline recommendations. GPs (*n* = 19) indicated a total of 84 barriers. Most GPs indicated barriers in (i) using the 4DSQ (*n* = 15), (ii) diagnosing anxiety and depressive disorders (*n* = 13) and (iii) allocating patients correctly to care, according to the severity of the disorder diagnosed (*n* = 15). Only some GPs perceived barriers in providing patient information (*n* = 5). The various barriers were classified according to the themes: knowledge and skills, attitude, time, patient’s opinion and behaviour, collaboration with mental health professionals and the availability of treatment. To address the barriers of each GP, various specific tailored interventions were delivered using two different formats, ‘peer group supervision’ and ‘personalised telephone consultation’. Two peer group supervisions, led by a GP of the research team, were attended by GP participants. The sessions lasted 2.5 h each. The supervisions focused on barriers relating to GPs’ knowledge and skills and perceptions of patient opinions and behaviours regarding diagnosis and treatment. Telephone consultations targeted the barriers relevant to individual GPs and covered GP knowledge and skills, perceptions of patient opinions and behaviours, time, attitude, collaboration with mental health professionals and local availability of evidence-based treatment options. Telephone consultations lasted 15 min and were provided once every 2 months by the interviewers for 1 year. Interviewers documented local implementation processes by making notes and offered advice to the GPs during the follow-up call. When GPs indicated that strategies for overcoming barriers were not successful, potential solutions were proposed to the GP during the next session. This dynamic feedback loop involving the interviewer and the GP was used in an attempt to maximise the effectiveness of the support offered. The identification of barriers to the implementation of guidelines, the development of interventions targeting these barriers and the application of the resulting tailored interventions has been described in detail [23].

### Outcomes

#### Clinical outcomes on practice level

To examine the effectiveness of tailored interventions to improve adherence to guidelines for anxiety and depression in general practice, the primary outcome was the proportion of recognised patients having anxiety or depression by the GP. Recognition was operationalised as the registration in the patients’ medical records, during 6 months preceding and after the EK-10 of terms describing (i) psychological complaints: anxiety, depression, worrying, sorrow or grief, stress, feeling down, disordered sleeping and unexplained somatic symptoms; (ii) the International Classification of Primary Care-1 (ICPC-1) codes [38] for anxiety, depression and related psychological problems, i.e. acute stress, feeling anger or irritation, behaving irritably or angrily, neurasthenia; or (iii) a completed 4DSQ.

In the preparation of the study in February 2010, before the data collection started, the primary outcome was changed (with approval of the funder ZonMw in March 2010), from an outcome on patient level (change in symptoms of anxiety and depression measured with the 4DSQ) to an outcome on the level of the cluster (recognition of anxiety and depression). The reason for this change was that the primary study aim focused on the performance of GP’s in the recognition of anxiety and depression.

Secondary outcomes at the cluster level were (i) number of consultations related to anxiety and depressive symptoms after recognition, (ii) prescription of antidepressants and (iii) referral to specialist mental healthcare. Data on cluster level outcomes were gathered by searching the patient medical records, from 6 months before until 6 months after completion of the EK-10. The search was performed by two researchers. To achieve a high inter-rater reliability, two researchers who were blind to the group assignment independently assessed 50 medical records, and weighted kappa statistics were calculated. A 5% sampling would be sufficient for a quality control [39]. The kappa for the primary outcome yielded an inter-rater agreement of 96% (weighted kappa = 0.91; 95% CI: 0.79–1.00). The kappas for the secondary outcomes yielded inter-rater agreements between 92% and 98% (weighted kappas were between 0.66 and 0.89).

#### Clinical outcomes on patient level

Secondary outcomes at the patient level were gathered using self-report questionnaires that were sent to the participants (via the Internet or by post) at baseline (T0), and 3 (T1) and 6 (T2) months later. These secondary outcome measures were used to evaluate the impact of clinical management.

Severity of anxiety and depressive symptoms was measured with the 4DSQ. The 4DSQ has four subscales relating to common psychopathology: distress, depression, anxiety and somatisation; high scores correspond to high symptom levels, and mean scores were calculated for all four subscales. The distress scale comprises 16 items; scores range from 0 to 32, a score ≥21 indicates a serious problem, such as clinically significant psychiatric disorder. The depression scale comprises six items; scores range from 0 to 12, a score ≥6 indicates probable depressive disorder. The anxiety scale comprises 12 items; scores range from 0 to 24, a score ≥9 indicates probable anxiety disorder. The somatisation scale comprises 16 items; scores range from 0 to 32, a score ≥21 indicates a somatic fixation.

Functional status was measured using the World Health Organisation’s Disability Assessment Scale II (WHODAS II) which covers functional impairments in six domains over the previous 30 days. The standardised total score, based on 32 or 36 items (36 only if work items are applicable), corrected for missing values was calculated [40,41]. The domains are communication and understanding, getting around, self-care, getting along with people, life activities (household and work) and participation in society. Scores range from 0 to 100; high scores indicated functional impairment.

Patients’ experience of GP provision of care for mental health problems was measured with the QUality Of care Through the Eyes (QUOTE) of the patient scale [42]. The QUOTE consists of six subscales measuring accessibility of care, providers’ emotional support, degree to which care is patient-centred, quality of care, provision of information and advice and guidance on self-help. Responses to items are measured on a four-point scale; high scores correspond to positive experiences.

### Sample size

The rate of recognition of anxiety or depression (primary outcome measure) was used for the power calculation and estimated at 45% [7,43]. Previous studies have shown that interventions targeting medical professionals’ adherence to guidelines can increase adherence by up to 10% [44]. Based on a review, we assumed that an intensive, tailored intervention of the type used in this study would improve recognition of anxiety and depression by as much as 15% [20]. To detect a 15% difference (60% versus 45%) (alpha = 0.05; power = 0.80) in recognition of anxiety and depression between the groups, assuming a 5% attrition rate (loss to follow-up was minimal because recognition was based on medical record review) and an intra-cluster correlation of 0.01 [45], we estimated that a sample size of 396 patients from 23 practices would be necessary. In an additional file, the covariates in the analyses are described [see Additional file 1].

### Statistical methods

Descriptive statistics were used to characterise the practices, GPs and patients. Comparisons between the intervention and control groups were made using *t* tests for continuous measures and *χ*^2^ tests for categorical variables (without imputation for missing values). These analyses were performed using the Statistical Package for the Social Sciences (SPSS) 19.0. Outcome variables were analysed with multilevel regression analyses taking into account the three-level clustering of observations (patients within GPs and GPs within practices) [46]. A fourth level of measurement, within-patients, was added as the lowest level for the analysis of the longitudinal data from self-report questionnaires at baseline and 3 and 6 months later. The type of regression model was matched to the outcome variable: logistic regression was used for dichotomous outcomes, linear regression for continuous outcomes and Poisson regression for number of consultations after recognition of symptoms of anxiety and depression.

Stata (version 12) was used for the multilevel regression analyses. The analyses were assessed according to intention to treat principles. Multiple imputation with chained equations (ICE) was used, creating 20 imputed datasets to compensate for missing values [47]. The imputation models included the primary and secondary outcome variables, group (intervention or control) and covariates according to the four-step strategy to select predictor variables described in van Buuren et. al. [48], namely patient gender and the following GP characteristics: gender, trainer status, psychologists working in the practice, attitude towards anxiety and depression, collaboration with a mental health nurse in primary care and level of burnout. Effect sizes were assessed for pretest and posttest effects using a control group design and standardised by pooled pretest standard deviations [49]. All analyses were prespecified.

## Results

### Participant flow and numbers analysed

Of 23 general practices recruited, 12 practices (23 GPs) were randomised to the intervention group and 11 practices (23 GPs) to the control group. Given the several recruitment strategies, it is not possible to present the exact recruitment rate. In the intervention group, 8 of 12 were rural practices, 4 practices were located in urban areas. In the control group four were rural practices and seven were located in urban areas. Compared to the Dutch distribution of GPs, it seems that rural practices are overrepresented [50]. All practices in both groups participated in the 1-day training session. Of all GPs, six GPs (26%) from three practices in the intervention group and four GPs (17%) from two practices in the control group did not participate. In all these cases, GPs working at the same practice promised to pass the information on to their absent colleagues. The consultation registration form was administered by 9 GPs (39%) from eight practices in the intervention group and 13 GPs (57%) from six practices in the control group; these GPs received individual feedback. GPs from 13 practices (7 in the intervention group) were contracted to a particular health insurance company and received the extra fee for each practice-listed patient in 2010 and 2011.

Five of seven GPs from one practice in the intervention group did not receive the tailored intervention; one became ill and the others state that they had insufficient time. In another practice, one of three GPs did not receive the tailored interventions because of a reported lack of time. Under the ‘intention to treat’, principle patients of all GPs were included in the study, except the patients of the GP who became ill because this GP saw no patients. One solo general practice was lost after 10 months because the GP emigrated; the patients of this GP were not excluded from the study.

**Figure 1:**
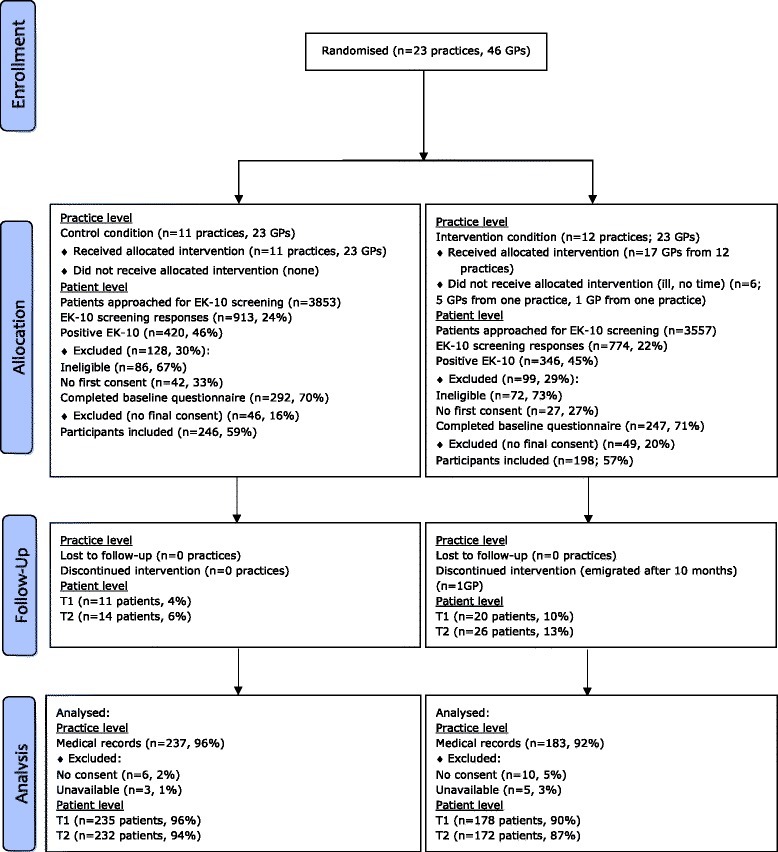
**Flow diagram of progress of clusters and individuals through the phases of the randomised trial.**

A total of 444 patients were included, 198 in the intervention group and 246 in the control group. Primary outcome data were obtained from 420 of 444 (95%) of the patients. Secondary outcome data were obtained from 413 of 444 (93%) of the patients at the 3-month follow-up (T1) and 404 of 444 (91%) at the 6-month follow-up (T2). Figure 1 shows the flow of general practices and patients. Regarding patients, the amount of missing information does not exceed 10%.

### Characteristics at baseline for clusters and individuals

Baseline characteristics for general practices, GPs and patients are given in Tables 1, 2 and 3, respectively.

**Table 1:** **Baseline characteristics of the intervention and control group given at cluster level**

**Variables**	**Intervention group**	**Control group**	***p*** **value**
**Practices**	***n*** **= 12**	***n*** **= 11**	
Practice type (%)			0.387
Solo	26.1	17.4	
Group	60.9	78.3	
Health centre	13.0	4.4	
Mean (SD) number of GPs in the practice	4.1 (2.9)	4.3 (2.4)	0.825
Disciplines working in the practice (%)			
Practice assistants	100	100	1.000
Practice nurses: somatic health services	95.7	100	0.312
Practice nurses: mental health services	65.2	39.1	0.007*
Physician assistants	0	13.0	0.073
Psychologists	0	4.4	0.312
Social workers	13.0	0	0.073
Physiotherapists	13.0	4.4	0.295
Others^a^	21.7	39.1	0.200
Mean (SD) number of patients per practice	5,476.3 (4,589.8)	5,393.5 (3,391.4)	0.945

*p value <0.05.

^a^Others: district nurse, assistant in training, dietician, pharmacist, speech therapist, podiatrist and psychologist specialising in mental health.

**Table 2:** **Baseline characteristics for GPs in the intervention and control group**

**Variables**	**Intervention group**	**Control group**	***p*** **value**
**General practitioners**	***n*** **= 23**	***n*** **= 23**	
Demographic characteristics			
Mean (SD) age (years)	49.5 (9.6)	47.7 (9.1)	0.511
Male (%)	65.2	43.5	0.139
Clinical experience, mean no. of years (SD)	18.4 (10.2)	16.4 (9.3)	0.500
Employment status (% ≥0.5 FTE)	95.7	100	0.312
Trainer status (% yes)	60.9	34.8	0.077
Interest and attitudes towards depressive and anxiety disorders			
Special interest in patients with anxiety and depressive disorders (% yes)	47.8	52.2	0.768
DAQ mean score (SD)			
Treatment attitudes	42.9 (5.6)	45.8 (8.3)	0.173
Professional unease	45.4 (8.2)	44.5 (7.4)	0.708
Depression malleability	38.4 (10.8)	41.8 (11.1)	0.302
Depression identification	47.6 (8.8)	45.7 (13.1)	0.560
REASON^a^ mean score (SD)			
Professional comfort with and competence in care of mental health problems	3.1 (0.4)	3.2 (0.5)	0.432
GPs’ concerns about problems with the health care system for treatment of anxiety and depression	4.5 (0.8)	4.3 (0.8)	0.641
Mean (SD) number of hours professional training on depression last year	2.8 (2.2)	3.4 (2.1)	0.408
Mean (SD) number of hours professional training on anxiety last year	2.0 (1.5)	2.5 (1.9)	0.290
Barriers to provision of health care^b^ (% score 4, 5 or 6)			
Perceived time limitations	65.2	60.9	0.760
Perceived lack of knowledge and skills for the recognition of anxiety and depressive disorders	13.0	13.0	1.000
Perceived lack of knowledge and skills for treatment of anxiety and depressive disorders	8.7	17.4	0.381
Barriers to implementation of guidelines^c^			
Barriers for implementation of depression and anxiety guidelines, mean score (SD)	34.0 (4.3)	33.8 (4.0)	0.859
Collaboration with professionals in mental health services (% yes)			
Mental health nurse in primary care	56.5	59.1	0.862
Primary care psychologist	13.0	21.7	0.437
Social workers	21.7	43.5	0.116
Specialist mental health worker	4.4	9.1	0.524
Levels of burnout (UBOS-C, %)			
Emotional exhaustion			0.038*
Low level	39.1	69.6	
Moderate level	61	30	
High level	-	-	
Depersonalisation			0.361
Low level	17.4	21.7	
Moderate level	65.2	73.9	
High level	17.4	4.4	
Personal accomplishment			0.741
Low level	17.4	26.1	
Moderate level	47.8	39.1	
High level	34.8	34.8	

*FTE* full-time equivalent, *DAQ* Depression Attitude Questionnaire, *UBOS-C* Utrecht Burn-Out Scale for the Contractual professions.

*p value <0.05.

^a^REASON questionnaire: GPs’ attitudes to their role in the management of anxiety and depressive disorders.

^b^Based on a questionnaire developed at the Scientific Institute for Quality of Health Care.

^c^The barriers and facilitators assessment instrument.

**Table 3:** **Baseline characteristics of the intervention and control group given at patient level**

**Variables**	**Intervention group**	**Control group**	***p*** **value**
**Patients**	***n*** **= 198** ^**b**^	***n*** **= 246** ^**c**^	
Sociodemographic characteristics			
Mean (SD) age (years)	53.4 (15.6)	55.0 (16)	0.294
Female	128 (64.7)	179 (72.8)	0.066
Born in the Netherlands	185 (95.4), *n* = 194	233 (95.5), *n* = 244	0.948
Married or living together	133 (68.6), *n* = 194	161 (65.7), *n* = 245	0.529
In paid employment	77 (42.3), *n* = 182	83 (34.7), *n* = 239	0.112
Level of education	*n* = 196	*n* = 244	0.647
Low	102 (52.0)	123 (50.4)	
Medium	48 (24.5)	69 (28.3)	
High	46 (23.5)	52 (21.3)	
Clinical characteristics			
Number of chronic medical conditions^a^ (range: 0–28), mean (SD)	3.1 (2.1)	3.0 (2.2)	0.723
4DSQ distress score (range: 0–32), mean (SD)	12.4 (8.1), *n* = 193	11.9 (7.1), *n* = 240	0.431
4DSQ depression score (range: 0–12), mean (SD)	1.8 (2.8), *n* = 194	1.6 (2.7), *n* = 239	0.480
4DSQ anxiety score (range: 0–24), mean (SD)	3.2 (4.0), *n* = 194	2.8 (3.5), *n* = 238	0.273
4DSQ somatisation score (range: 0–32), mean (SD)	8.5 (5.9), *n* = 186	8.3 (5.9), *n* = 228	0.760

Values are numbers (percentages) unless stated otherwise.

*4DSQ* Four-Dimensional Symptom Questionnaire.

^a^Chronic medical condition was measured with the Dutch Central Bureau of Statistics (CBS) list.

^b^ *n* = 198 unless stated otherwise.

^c^ *n* = 246 unless stated otherwise.

### Primary outcome measure

The proportion of patients with adequately recognised and documented anxiety or depression was significantly higher in the intervention group than the control group (42% versus 31%). In the intervention group, it was more likely that anxiety and depression would be recognised (odds ratio = 1.60; 95% CI: 1.01–2.53; *p* = 0.047; see Table 4). The intra-cluster correlation for the combined level of general practitioners and practices was 0.02. In an additional file, the cost per one additional recognised patient is described [see Additional file 2].

**Table 4:** **Odds ratios for the variable recognition (primary outcome), prescribing antidepressants, referral to specialist mental health services and number of consultations after recognition (secondary outcomes), using mixed effects (logistic or Poisson) regression models on multiple imputed data**

**Outcome variable**	**Odds ratio**	**95% confidence interval**	**Test statistics and** ***p*** **value**	**Estimated proportion in control group**	**Estimated proportion in intervention group**
Recognition (primary outcome)	1.60*	(1.01, 2.53)	*z* = 1.99, *p* = 0.047	0.31	0.42
Number of consultations	1.78*	(1.14, 2.78)	*z* = 2.52, *p* = 0.012	0.57^a^	1.01^a^
Prescribing antidepressants	1.07	(0.52, 2.19)	*z* = 0.19, *p* = 0.849	0.12	0.13
Referral to specialist mental health services	1.62	(0.72, 3.64)	*z* = 1.16, *p* = 0.247	0.05	0.08

All mixed effects models were estimated on multiple imputed data (20 datasets) having a multilevel data structure of 444 patients (lowest level) of 40 general practitioners (second level) of 23 practices (highest level).

*p value <0.05.

^a^For ‘number of consultations’, the incidence rate ratio (IRR) and incidence rates from the Poisson regression are displayed instead of an odds ratio (OR) and probabilities.

### Secondary outcome measures

There were no significant differences between the groups for most of the secondary outcome variables (proportion of patients who were prescribed antidepressants: odds ratio (OR) = 1.07, 95% CI: 0.52–2.19; proportion of patients who were referred to specialist mental health services: OR = 1.62, 95% CI: 0.72–3.64). In contrast, patients whose symptoms of depression and anxiety were recognised received significantly more frequent consultations if their GP was in the intervention group (mean number of consultations 1.06) than if their GP was in the control group (mean number of consultations 0.64) (incidence rate ratio 1.78, 95% CI: 1.14–2.78). Table 4 provides a detailed description of these data.

Table 5 shows the secondary outcome data at patient level: scores on the 4DSQ and WHODAS II. There were no significant differences between the groups on the 4DSQ distress, anxiety and somatisation subscales; however, patients in the intervention group showed a significant additional reduction in depressive symptoms at the 3-month posttest (T1, effect size (ES) = 0.2; *p* < 0.05).

**Table 5:** **Predicted mean of the 4DSQ and WHODAS II scores and effect size of patients from GPs receiving training, feedback and tailored interventions (intervention group,** ***N*** **= 198) or training and feedback (control group,** ***N*** **= 246) based on multilevel linear regression analysis on imputed data, from baseline to 3 and 6 months**

	**Group**	**Baseline**		**3 months**				**6 months**			
		**Mean**	**95% CI**	**Mean**	**95% CI**	**ES**	***p*** **value**	**Mean**	**95% CI**	**ES**	***p*** **value**
Distress	Intervention	12.37	(11.08, 13.66)	11.16	(9.77, 12.56)	−0.0	0.912	10.49	(9.10, 11.87)	−0.1	0.235
	Control	11.86	(10.63, 13.09)	10.72	(9.48, 11.96)			10.76	(9.50, 12.01)		
Depression	Intervention	1.80	(1.40, 2.21)	1.27	(0.85, 1.69)	−0.2*	0.027	1.48	(1.04, 1.91)	−0.1	0.445
	Control	1.58	(1.20, 1.97)	1.52	(1.14, 1.91)			1.42	(1.03, 1.81)		
Anxiety	Intervention	3.14	(2.51, 3.78)	2.65	(2.01, 3.30)	−0.0	0.847	2.38	(1.73, 3.03)	−0.1	0.141
	Control	2.77	(2.15, 3.40)	2.33	(1.71, 2.96)			2.40	(1.78, 3.03)		
Somatisation	Intervention	8.51	(7.61, 9.41)	8.58	(7.65, 9.52)	+0.1	0.284	8.74	(7.78, 9.69)	−0.1	0.065
	Control	8.25	(7.41, 9.09)	7.85	(7.00, 8.70)			7.61	(6.75, 8.48)		
WHODAS II	Intervention	25.82	(23.59, 28.06)	24.32	(22.01, 26.63)	+0.1	0.245	21.41	(19.07, 23.75)	−0.1	0.293
	Control	23.57	(21.57, 25.57)	20.68	(18.65, 22.71)			20.39	(18.36, 22.42)		

*4DSQ* Four-Dimensional Symptom Questionnaire, *WHODAS II* World Health Organisation’s Disability Assessment Scale II, *ES* effect size of pretest-posttest-control group design using pooled pretest standard deviation.

*p < 0.05, where *p* is the significance level of the group × time interaction term.

Table 6 shows the data on patients’ perceptions of the care they received from their GP for mental health problems. Patients in the intervention group reported significantly more positive experiences of the accessibility of care (ES = 0.4; *p* < 0.05) and better provision of information and advice (ES = 0.5; *p* < 0.05) at 6 months than patients in the control group. There were no significant differences between the intervention group and control group in the other four domains (providers’ emotional support, degree to which care is patient-centred, quality of care, guidance on self-help).

**Table 6:** **Predicted mean QUOTE scores and effect size of patients from GPs receiving training, feedback and tailored interventions (intervention group,** ***N*** **= 198) or training and feedback (control group,** ***N*** **= 246) based on multilevel linear regression analysis on imputed data, from baseline to 3 and 6 months**

	**Group**	**Baseline**		**3 months**				**6 months**			
		**Mean**	**95% CI**	**Mean**	**95% CI**	**ES**	***p*** **value**	**Mean**	**95% CI**	**ES**	***p*** **value**
Accessibility of care	Intervention	3.68	(3.45, 3.92)	3.61	(3.35, 3.87)	+0.2	0.263	3.72	(3.44, 4.00)	+0.4*	0.038
	Control	3.49	(3.25, 3.73)	3.25	(2.98, 3.51)			3.19	(2.92, 3.46)		
Emotional support	Intervention	3.04	(2.86, 3.22)	3.05	(2.84, 3.25)	+0.2	0.341	3.21	(2.99, 3.44)	+0.3	0.143
	Control	3.10	(2.93, 3.27)	2.96	(2.76, 3.16)			3.04	(2.83, 3.24)		
Patient-centred care	Intervention.	3.29	(3.14, 3.44)	3.21	(3.04, 3.38)	+0.2	0.294	3.31	(3.12, 3.50)	+0.1	0.642
	Control	3.28	(3.14, 3.42)	3.07	(2.91, 3.23)			3.23	(3.07, 3.40)		
Quality of care	Intervention	2.84	(2.66, 3.02)	2.85	(2.64, 3.06)	+0.1	0.599	3.03	(2.81, 3.26)	+0.1	0.525
	Control	2.86	(2.68, 3.04)	2.80	(2.60, 3.00)			2.96	(2.75, 3.16)		
Information and advice	Intervention	3.21	(3.03, 3.38)	3.12	(2.92, 3.32)	+0.0	0.842	3.48	(3.26, 3.70)	+0.5*	0.013
	Control	3.29	(3.12, 3.45)	3.17	(2.98, 3.37)			3.18	(2.98, 3.38)		
Self-help advice	Intervention	2.77	(2.57, 2.97)	2.85	(2.62, 3.09)	+0.1	0.649	2.99	(2.73, 3.24)	+0.2	0.311
	Control	2.86	(2.67, 3.06)	2.87	(2.64, 3.09)			2.89	(2.66, 3.12)		

*QUOTE* QUality Of care Through the Eyes of the patient, *ES* effect size of pretest-posttest-control group design using pooled pretest standard deviation.

*p < 0.05, where *p* is the significance level of the group × time interaction term.

## Discussion

### Statement of principal findings

The results of this study indicate that a tailored intervention was significantly more effective in improving recognition of anxiety and depression in general practice than training and feedback alone. GPs in the intervention group had more frequent consultations with patients whose anxiety and depression had been recognised than GPs in the control group, although this did not lead to more frequent prescription of antidepressants or referral to specialist mental health services. In addition, patients of GPs in the intervention group showed an additional reduction in depressive symptoms at 3 months compared with patients in the control group. However, no significant difference in depressive symptoms was found at 6 months. Patients of GPs in the intervention group also reported significantly more positive experiences of the accessibility of care and better provision of information and advice than patients of GPs in the control group. No significant differences between the groups were found on the other secondary outcomes.

### Strengths and limitations

The use of a systematic intensive tailored intervention to improve the recognition diagnosis and treatment of anxiety and depression in general practice was an important strength of this study. The study also had some limitations. Firstly, the primary outcome was the recognition by GPs of anxiety or depression in patients who had screened positive on the EK-10. The EK-10 is the preferred instrument for screening for anxiety and depressive disorders in general practice; however, about one third of all patients who screened positive on the EK-10 had low 4DSQ scores, indicating that they had few symptoms and were in fact not suffering from anxiety or depression. The EK-10 has a specificity of 0.75 for detecting any depressive and/or anxiety disorder. As a consequence, patients may be included while they did not have an anxiety or depressive disorder. Given the recruitment strategy used in our study (all adult patients from the included general practices who visited their GP) and a low mean score on the 4DSQ for patients screening positive on EK-10, the subgroup of patients with mild symptoms might have affected the rate of GP recognition of anxiety and depression and the impact of clinical management. Besides, assuming that the population only included positive screens, it is not clear whether the intervention led to over recognition and false positives. Besides, the relatively small number of patients who could be screened (compared to the large numbers who were approached for screening) and that almost half of these patients screened positive might indicate selection bias. Secondly, patients started to enter the study about 4 months after the start of the tailored intervention. The intervention may have been more effective for patients when they were included after the tailored intervention was finished, because at this point, GPs would have been exposed to the intervention for longer and would have had opportunity to change their professional practice. Thirdly, six GPs in the intervention group who did not receive the tailored intervention were included due to the intention to treat principle; the patients of these GPs received ‘treatment as usual’, i.e. the same treatment as the control group, diluting potential differences between the conditions.

Finally, the effect of the intervention on patient outcomes is unclear, because the study focused on GP performance.

### Comparison with existing literature

A previous RCT compared the effectiveness in overcoming obstacles to change in the implementation of guidelines for depression in general practice of a tailored intervention based on psychological theories and simple dissemination of the guidelines [22]. The tailored intervention was delivered once and results showed that it increased adherence to some of the guideline recommendations. In our study, the tailored intervention included provision of one-to-one telephone consultations every 2 months for 1 year. This enabled the provision of dynamic feedback; whenever strategies for overcoming barriers did not appear to be succeeding, new solutions were developed and discussed with the GP during the next consultation. It was hoped that this dynamic feedback loop would maximise the effectiveness of the tailoring process. In another RCT, the effectiveness of an intervention to reduce barriers to GPs’ adherence to recommended standards for recognition and management of late-life depression was assessed [21]. GPs in the intervention group received patient-specific treatment recommendations in three special visits; GPs in the control group received no intervention. The results showed that GPs in the intervention group were more likely to diagnose depression and prescribe antidepressants, but no effect on patient outcomes was detected. In this study, all GPs received the same intervention, unlike our study where the specific content of the intervention was tailored to the individual GP.

A systematic review of tailored interventions targeting identified barriers to change concluded that tailored interventions are more likely to improve professional practice than no intervention or simple dissemination of guidelines. Our study indicates that training, feedback and a tailored intervention are more effective than training and feedback. However, reviews have shown that educational meetings and feedback can also improve professional practice [24,25].

### Interpretation of the findings and implications for future research

Tailored interventions have been suggested to be a promising approach to improve adherence to guidelines relating to recognition and management of anxiety and depression in general practice. Our study adds to the evidence supporting the use of such interventions. However, despite the increased recognition and the provision of more consultations by GPs in the intervention group, no effect was seen on the reduction of symptoms and improvement of functional status. Possible explanations can be the following: the effect of treatment is too small to establish, GPs’ treatment cannot improve the natural course and the treatment is not effective.

In our study, patients with minor symptoms of anxiety and depression were included. Future research could focus on determining the effectiveness of the tailored intervention for patients presenting with more severe symptoms of anxiety and depression in primary care.

Tailored interventions may be a solution to improve the uptake of guideline recommendations for anxiety and depression in general practice, but more research is needed before large-scale tailored implementation can be recommended. Also, the cost-effectiveness of the tailored intervention should be studied.

## Conclusions

We described the effect of a tailored intervention on adherence to recommended diagnostic procedures and treatment for patients with suspected anxiety and depression. Our study showed that a tailored implementation programme may enhance the recognition of patients with anxiety or depression. Further development of the programme is advisable and to determine the cost-effectiveness.

## Additional files

Additional file 1:
**The covariates in the analyses.** This file provides insight in and results of the procedure which was used for the identification of possible covariates. Additional file 2:
**The cost per one additional recognised patient.** This file provides the results of an economic evaluation.

## References

[1] Demyttenaere K, Bruffaerts R, Posada-Villa J, Gasquet I, Kovess V, Lepine JP (2004). Prevalence, severity, and unmet need for treatment of mental disorders in the World Health Organization World Mental Health Surveys. JAMA.

[2] Alonso J, Angermeyer MC, Bernert S, Bruffaerts R, Brugha TS, Bryson H, de GG, Graaf R, Demyttenaere K, Gasquet I (2004). Disability and quality of life impact of mental disorders in Europe: results from the European Study of the Epidemiology of Mental Disorders (ESEMeD) project. Acta Psychiatr Scand Suppl.

[3] Gustavsson A, Svensson M, Jacobi F, Allgulander C, Alonso J, Beghi E (2011). Cost of disorders of the brain in Europe 2010. Eur Neuropsychopharmacol.

[4] Verhaak PF, van Dijk CE, Nuijen J, Verheij RA, Schellevis FG (2012). Mental health care as delivered by Dutch general practitioners between 2004 and 2008. Scand J Prim Health Care.

[5] van Avendonk M, van Weel-Baumgarten E, van der Weele G, Wiersma T, Burgers JS (2012). Summary of the Dutch College of General Practitioners’ practice guideline ‘Depression’. Ned Tijdschr Geneeskd.

[6] van Avendonk MJ, Hassink-Franke LJ, Terluin B, van Marwijk HW, Wiersma T, Burgers JS (2012). Summarisation of the NHG practice guideline ‘anxiety’. Ned Tijdschr Geneeskd.

[7] Lecrubier Y (2007). Widespread underrecognition and undertreatment of anxiety and mood disorders: results from 3 European studies. J Clin Psychiatry.

[8] Piek E, Nolen WA, van der Meer K, Joling KJ, Kollen BJ, Penninx BW (2012). Determinants of (non-) recognition of depression by general practitioners: results of the Netherlands Study of Depression and Anxiety. J Affect Disord.

[9] Fernandez A, Haro JM, Martinez-Alonso M, Demyttenaere K, Brugha TS, Autonell J (2007). Treatment adequacy for anxiety and depressive disorders in six European countries. Br J Psychiatry.

[10] Smolders M, Laurant M, Verhaak P, Prins M, van Marwijk H, Penninx B (2009). Adherence to evidence-based guidelines for depression and anxiety disorders is associated with recording of the diagnosis. Gen Hosp Psychiatry.

[11] Prins MA, Verhaak PF, Smolders M, Laurant MG, van der Meer K, Spreeuwenberg P (2010). Patient factors associated with guideline-concordant treatment of anxiety and depression in primary care. J Gen Intern Med.

[12] Adli M, Bauer M, Rush AJ (2006). Algorithms and collaborative-care systems for depression: are they effective and why? A systematic review. Biol Psychiatry.

[13] Andrews G, Issakidis C, Sanderson K, Corry J, Lapsley H (2004). Utilising survey data to inform public policy: comparison of the cost-effectiveness of treatment of ten mental disorders. Br J Psychiatry.

[14] Verhaak P, Prins MA, Spreeuwenberg P, Draisma S, van Balkom T, Bensing JM (2009). Receiving treatment for common mental disorders. Gen Hosp Psychiatry.

[15] Wittkampf KA, van Zwieten M, Smits FT, Schene AH, Huyser J, van Weert HC (2008). Patients’ view on screening for depression in general practice. Fam Pract.

[16] Tylee A, Walters P (2007). Underrecognition of anxiety and mood disorders in primary care: why does the problem exist and what can be done?. J Clin Psychiatry.

[17] Barley EA, Murray J, Walters P, Tylee A (2011). Managing depression in primary care: a meta-synthesis of qualitative and quantitative research from the UK to identify barriers and facilitators. BMC Fam Pract.

[18] Smolders M, Laurant M, van Wamel A, Grol R, Wensing M (2008). What determines the management of anxiety disorders and its improvement?. J Eval Clin Pract.

[19] van Dijk CE, Verheij RA, Spreeuwenberg P, van den Berg MJ, Groenewegen PP, Braspenning J (2013). Impact of remuneration on guideline adherence: empirical evidence in general practice. Scand J Prim Health Care.

[20] Baker R, Camosso-Stefinovic J, Gillies C, Shaw E, Cheater F, Flottorp S, Robertson N (2010). Tailored interventions to overcome identified barriers to change: effects on professional practice and health care outcomes. Cochrane Database Of Systematic Reviews (Online).

[21] Callahan CM, Hendrie HC, Dittus RS, Brater DC, Hui SL, Tierney WM (1994). Improving treatment of late life depression in primary care: a randomized clinical trial. J Am Geriatr Soc.

[22] Baker R, Reddish S, Robertson N, Hearnshaw H, Jones B (2001). Randomised controlled trial of tailored strategies to implement guidelines for the management of patients with depression in general practice. Br J Gen Pract.

[23] Sinnema H, Terluin B, Wensing M, Volker D, Franx G, van Balkom A (2013). Systematic tailoring for the implementation of guideline recommendations for anxiety and depressive disorders in general practice: perceived usefulness of tailored interventions. BMC Fam Pract.

[24] Forsetlund L, Bjorndal A, Rashidian A, Jamtvedt G, O’Brien MA, Wolf F, Davis D, Odgaard-Jensen J, Oxman AD (2009). Continuing education meetings and workshops: effects on professional practice and health care outcomes. Cochrane Database Syst Rev.

[25] Ivers N, Jamtvedt G, Flottorp S, Young JM, Odgaard-Jensen J, French SD (2012). Audit and feedback: effects on professional practice and healthcare outcomes. Cochrane Database Syst Rev.

[26] Sinnema H, Franx G, Volker D, Majo C, Terluin B, Wensing M (2011). Randomised controlled trial of tailored interventions to improve the management of anxiety and depressive disorders in primary care. Implement Sci.

[27] Donker T, Comijs H, Cuijpers P, Terluin B, Nolen W, Zitman F (2010). The validity of the Dutch K10 and extended K10 screening scales for depressive and anxiety disorders. Psychiatry Res.

[28] Terluin B, van Heest F, van der Meer K, Neomagus G, Hekman J, Aulbers L (2004). Dutch College of General Practitioners guideline: anxiety disorder, first revision [NHG-Standaard Angststoornissen, eerste herziening. In Dutch]. Huisarts en Wetenschap.

[29] Richtlijnwerkgroep Multidisciplinaire richtlijnen Angststoornissen en Depressie (2009). Multidisciplinary guideline anxiety disorders: guideline for diagnostics and treatment of adult clients with an anxiety disorder, first revision [Multidisciplinaire richtlijn Angststoornissen.

[30] Van Marwijk HWJ, Grundmeijer HGLM, Bijl D, Van Gelderen MG, De Haan M, Van Weel-Baumgarten EM (2003). Dutch College of General Practitioners guideline: depression, first revision [NHG-Standaard Depressieve stoornis (depressie). Eerste herziening. In Dutch]. Huisarts Wetenschap.

[31] Terluin B, van Marwijk HW, Ader HJ, de Vet HC, Penninx BW, Hermens ML (2006). The Four-Dimensional Symptom Questionnaire (4DSQ): a validation study of a multidimensional self-report questionnaire to assess distress, depression, anxiety and somatization. BMC Psychiatry.

[32] Haaga DA (2000). Introduction to the special section on stepped care models in psychotherapy. J Consult Clin Psychol.

[33] Katon W, von Korff M, Lin E, Simon G, Walker E, Unutzer J (1999). Stepped collaborative care for primary care patients with persistent symptoms of depression: a randomized trial. Arch Gen Psychiatry.

[34] Von Korff M, Tiemens B (2000). Individualized stepped care of chronic illness. West J Med.

[35] van Rijswijk E, van Hout H, van de Lisdonk E, Zitman F, van Weel C (2009). Barriers in recognising, diagnosing and managing depressive and anxiety disorders as experienced by family physicians; a focus group study. BMC Fam Pract.

[36] Henke RM, Chou AF, Chanin JC, Zides AB, Scholle SH (2008). Physician attitude toward depression care interventions: implications for implementation of quality improvement initiatives. Implement Sci.

[37] Smith L, Walker A, Gilhooly K (2004). Clinical guidelines of depression: a qualitative study of GPs’ views. J Fam Pract.

[38] Lamberts HWM (1990). International Classification of Primary Care (ICPC).

[39] Liddy C, Wiens M, Hogg W (2011). Methods to achieve high interrater reliability in data collection from primary care medical records. Ann Fam Med.

[40] Chwastiak LA, Von Korff M (2003). Disability in depression and back pain Evaluation of the World Health Organization Disability Assessment Schedule (WHO DAS II) in a primary care setting. J Clin Epidemiol.

[41] Ustun TB, Chatterji S, Kostanjsek N, Rehm J, Kennedy C, Epping-Jordan J (2010). Developing the World Health Organization Disability Assessment Schedule 2.0. Bull World Health Organ.

[42] Sixma HJ, Kerssens JJ, van Campen C, Peters L (1998). Quality of care from the patients’ perspective: from theoretical concept to a new measuring instrument. Health Expect.

[43] Hickie IB, Davenport TA, Scott EM, Hadzi-Pavlovic D, Naismith SL, Koschera A (2001). Unmet need for recognition of common mental disorders in Australian general practice. Med J Aust.

[44] Grimshaw JM, Thomas RE, MacLennan G, Fraser C, Ramsay CR, Vale L, Whitty P, Eccles MP, Matowe L, Shirran L (2004). Effectiveness and efficiency of guideline dissemination and implementation strategies. Health Technol Assess.

[45] Adams G, Gulliford MC, Ukoumunne OC, Eldridge S, Chinn S, Campbell MJ (2004). Patterns of intra-cluster correlation from primary care research to inform study design and analysis. J Clin Epidemiol.

[46] Twisk JWR (2006). Applied multilevel analysis.

[47] Royston P, White IR (2011). Multiple Imputation by Chained Equations (MICE): implementation in stata. J Stat Softw.

[48] van Buuren S, Boshuizen HC, Knook DL (1999). Multiple imputation of missing blood pressure covariates in survival analysis. Stat Med.

[49] Morris SB (2008). Estimating effect sizes from pretest-posttest-control group designs. Organ Res Methods.

[50] Hingstman L, Kenens RJ (2010). Figures from the registration of general practitioners: survey 2010 [in Dutch].

