# Supplementary material for: Effectiveness of a tailored implementation programme to improve recognition, diagnosis and treatment of anxiety and depression in general practice: a cluster randomised controlled trial
Source: Implement Sci. 2015 Mar 12;10:33. doi: 10.1186/s13012-015-0210-8 (PMC4360947; doi:10.1186/s13012-015-0210-8)
Supplement: Additional file 1: — The covariates in the analyses. This file provides insight in and results of the procedure which was used for the identification of possible covariates. [file 13012_2015_210_MOESM1_ESM.docx]

**Effectiveness of a tailored implementation programme to improve recognition, diagnosis and treatment of anxiety and depression in general practice: a cluster randomised controlled trial**

Henny Sinnema, *Research Associate*^1^, Maria Cristina Majo *Research Associate*^1^, Daniëlle Volker, *Research Associate*^1^, Adriaan Hoogendoorn, *Senior Researcher*^2^, Berend Terluin *Senior Researcher*^3^, Michel Wensing, ***Professor*** *of Implementation Science*^4^, Anton van Balkom, ***Professor of*** *Evidence-based* ***Psychiatry*^2^**

^1^ Netherlands Institute of Mental Health and Addiction, Trimbos Institute, 3500 AS Utrecht, The Netherlands

^2^ Department of Psychiatry, VU University Medical Centre and GGZinGeest, 1081 HL Amsterdam, The Netherlands

^3^ Department of General Practice and Elderly Care Medicine, EMGO Institute for Health and Care Research, 1000 SN Amsterdam, The Netherlands

^4^ Radboud University Medical Centre, Scientific Institute for Quality, 6500 HB Nijmegen, The Netherlands

Correspondence to: H Sinnema [hsinnema@trimbos.nl](mailto:hsinnema@trimbos.nl)

Additional file 1 Covariates in the analyses

Owing to the small number of clusters it was possible that the randomisation procedure would produce systematic group differences, we therefore collected data on a range of GP and patient characteristics (Tables 1, 2, 3) that might be related to reported outcomes. All these data were self-reported.

GPs’ attitudes to anxiety and depression were measured with the Depression Attitude Questionnaire (DAQ) [1]and with the REASON questionnaire.[2] The DAQ measures GPs’ interest in and attitudes to depressive and anxiety disorders; respondents are asked to indicate the degree to which they agree or disagree with statements based on their day-to-day clinical experience. The DAQ consists of subscales: treatment attitude (high scores indicate a preference for antidepressant drugs, low scores for psychotherapy); professional unease (high scores indicate discomfort in dealing with depressed patients, perception that treating depression is unrewarding and that patients would be better off being managed by a specialist); depression malleability (high scores indicate pessimism about one’s ability to modify the course of depression) and depression identification (high scores indicate difficulty in differentiating depression from unhappiness and that there is likely to be little additional benefit beyond a GP's own treatment. The REASON measures GPs' attitudes to their role in the management of patients with depressive and anxiety disorders, and comprises two subscales: (i) professional comfort with and competence in care of mental health disorders (low scores indicate comfort and competence) and (ii) GPs' concerns about problems with the health care system for management of anxiety and depression (low scores indicate concerns about difficulties).

Barriers to providing effective healthcare for patients with depressive or anxiety disorders were assessed by three items based on a previously developed questionnaire.[3] Responses were measured on a six-point Likert scale ranging from 1 (not at all) to 6 (to a very great extent). Perceived barriers to implementation of the depression and anxiety guidelines were measured with a barriers and facilitators assessment instrument[4] which asks respondents to rate items on a five-point Likert scale, ranging from 1 (completely disagree) to 5 (completely agree); a total score was calculated by summing the scores for individual items. GPs' levels of burnout were measured using the Utrecht Burn-Out Scale for the Contactual professions (UBOS-C).[5] The UBOS-C comprises 20 items divided into three subscales: emotional exhaustion; depersonalisation and personal accomplishment.

We used four criteria to estimate possible differences between the groups in terms of GP characteristics (cluster level) and patient characteristics (individual level): (1) the variable is a possible confounder based on previous research or theory; (2) the intervention group and the control group demonstrated substantial differences at baseline with regard to this variable, regardless of the *p*-value of the difference; (3) the variable was substantially associated with the outcome measurement at follow-up (high predictive value); (4) the effect size of the intervention changed substantially (Cohen’s d > 0.2, corresponding to an odds ratio > 1.44)[6] after inclusion of the variable in the regression model, regardless of the *p*-value. Any variable which fulfilled all four criteria would be defined as a covariate. No covariates were identified using this procedure.

**References**

1. Botega N, Blizard R, Wilkinson G: **General practitioners and depression-first use of the depression attitude questionnaire.** *International Journal of Methods in Psychiatric Research* 1992, **4:**169-180.

2. McCall L, Clarke DM, Rowley G: **A questionnaire to measure general practitioners' attitudes to their role in the management of patients with depression and anxiety.** *Aust Fam Physician* 2002, **31:**299-303.

3. Heideman J, Lin van N, Laurant M, Akkermans R, Drenthen T, Grol R: **Determinants of general practitioners' practices regarding mental illness [in Dutch].** *Tijdschrift voor Gezondheidswetenschappen* 2005, **83:**342-348.

4. Peters MAJ, Harmsen M, Laurant MGH, Wensing M: *Room for improvement? Barriers to and facilitators for improvement in patient care.* Nijmegen: Centre for Quality of Care Research; 2003.

5. Schaufeli WB, Dierendonk van D: *UBOS (Utrechtse Burnout Schaal). Handleiding.* Lisse: Swets & Zeitlinger; 2000.

6. Chinn S: **A simple method for converting an odds ratio to effect size for use in meta-analysis.** *Stat Med* 2000, **19:**3127-3131.
