# Supplementary material for: Effectiveness of a tailored implementation programme to improve recognition, diagnosis and treatment of anxiety and depression in general practice: a cluster randomised controlled trial
Source: Implement Sci. 2015 Mar 12;10:33. doi: 10.1186/s13012-015-0210-8 (PMC4360947; doi:10.1186/s13012-015-0210-8)
Supplement: Additional file 2: — The cost per one additional recognised patient. This file provides the results of an economic evaluation. [file 13012_2015_210_MOESM2_ESM.docx]

**Effectiveness of a tailored implementation programme to improve recognition, diagnosis and treatment of anxiety and depression in general practice: a cluster randomised controlled trial**

Henny Sinnema, *Research Associate*^1^, Maria Cristina Majo *Research Associate*^1^, Daniëlle Volker, *Research Associate*^1^, Adriaan Hoogendoorn, *Senior Researcher*^2^, Berend Terluin *Senior Researcher*^3^, Michel Wensing, ***Professor*** *of Implementation Science*^4^, Anton van Balkom, ***Professor of*** *Evidence-based* ***Psychiatry*^2^**

^1^ Netherlands Institute of Mental Health and Addiction, Trimbos Institute, 3500 AS Utrecht, The Netherlands

^2^ Department of Psychiatry, VU University Medical Centre and GGZinGeest, 1081 HL Amsterdam, The Netherlands

^3^ Department of General Practice and Elderly Care Medicine, EMGO Institute for Health and Care Research, 1000 SN Amsterdam, The Netherlands

^4^ Radboud University Medical Centre, Scientific Institute for Quality, 6500 HB Nijmegen, The Netherlands

Correspondence to: H Sinnema [hsinnema@trimbos.nl](mailto:hsinnema@trimbos.nl)

**Additional file 2: the cost per one additional recognised patient**

Costs

An economic evaluation was conducted to estimate the cost effectiveness of the tailored intervention from a societal perspective. Cost-effectiveness was determined by calculating the cost of medical treatment, costs associated loss of productivity using the Trimbos institute Medical Technology Assessment questionnaire for Costs associated with Psychiatric Illness (TiC-P).[1] Data were collected at baseline (T0), and 3 (T1) and 6 (T2) months later. An incremental cost effectiveness ratio was calculated to determine the cost per one additional recognized patient.

The TiC-P covers 3 cost categories: direct medical costs, direct non-medical costs, and indirect non-medical costs. Direct medical costs relate to the utilisation of health care services. To calculate these costs, health service units were multiplied by the standard full economic cost given in the Dutch guidelines for economic evaluations of healthcare for 2010 .[2] Costs for prescription of psychotropic drugs (e.g. antidepressants, benzodiazepines and antipsychotics) were calculated from price per standard daily dose given in the Dutch Pharmacotherapeutic Compass, the number of prescription days and pharmacists’ dispensing costs (assumed to be €14 per prescription).[3]

Direct non-medical costs encompassed the cost of patient travel to receive professional help and loss of leisure time (valued at €12.66 per hour)[2] and informal caregivers’ time e.g. friends, neighbours, family running errands for participants (valued at €12.66 per hour) (Table 1). The cost of loss of productivity (indirect non-medical costs) was calculated based on days of absence from paid work (absenteeism) and an estimate of days working with reduced efficiency (presenteeism).[2]

The incremental cost effectiveness ratio was calculated by dividing incremental costs by incremental effects. Cost-utility and the per-participant intervention cost were estimated. Intervention costs comprised: fees for the interviewers and researchers’ activities during the tailoring process, the one-day training session and provision of feedback; material costs for the one-day training session and cost of GP time for the one-day training session, interviews, peer group supervisions and telephone consultations. Researchers activities were added because when the tailored intervention is implemented in practice, these costs will be incurred by the person who will identify barriers and tailor interventions. The intervention costs were different for the intervention and control groups.

Cost effectiveness analyses

The cost-effectiveness analyses were conducted using Stata version 11 (StataCorp LP, College Station, Texas, USA) and Excel. Missing values for medical costs and quality of life scores were imputed using multiple imputation. Twenty imputed datasets were created with SPSS version 20 using fully a conditional specification and predictive mean matching. The mean total costs per participant per month for each of the groups were calculated at baseline (T0), after 3 months (T1) and at the 6-month follow-up (T2). Incremental costs were calculated as the between-group difference at follow-up (6 months).

Both incremental costs and incremental effects were used to calculate the ICER. The ICER was calculated as (*C*_1_‑ *C*_0_)/(*E*_1_‑ *E*_0_), where *C* is the average per patient cost over a 6-month time frame, *E* is the proportion of patients who were recognised by the GP having anxiety or depression and the subscript indicates the group (0 = control group). The ICER gives the incremental cost of recognising depression or anxiety in one extra patient.

Non-parametric bootstrapping in Excel was used to simulate 2500 ICERs that were plotted on the cost-effectiveness plane. In this way, the degree of uncertainty associated with the ICER was captured.

Sensitivity Analysis

The accuracy of the estimated per-participant costs is uncertain. To ascertain the robustness of the overall findings the analyses were repeated for four alternative scenarios: (i) excluding the patients with extreme values (outliers) for monthly total cost at baseline groups (exploratory, not pre-specified); (ii) the highest mean total cost (pre-specified), (iii) the lowest mean total cost (pre-specified), and (iv) excluding patients’ from the 6 GPs in the intervention group who did not receive the tailored intervention (not pre-specified). These scenarios were selected because substantial differences between them might affect the final conclusion.

Economic evaluation

Table 2 shows the costs associated with the various components of the control and intervention groups at baseline and at the 6-month follow-up. At baseline, the mean total cost per participant per month was €730.85 (SD 1147.07) in the control group and €987.08 (SD 1596.15) in the intervention group; *p*=0.06. The main difference between the groups at baseline was in costs associated with loss of productivity.

The mean total cost per participant over a 6-month time frame was estimated to be €1,734 in the control group and €2,463.45 in the intervention group, giving an incremental cost per participant per 6-months of €729=€2,463.45-€1,734(rounded to the nearest Euro).

The difference in effectiveness was 0.42 – 0.31 = 0.11 (incremental effectiveness), where 42% of the patients were recognized in the intervention group compared to 31% in the control group. Giving an estimated mean ICER of €6,807=€729/0.11 (after rounding) for recognition of anxiety or depression in one additional patient (95% CI: €-273,002 - €336,608). Using the 2500 bootstraps, the median ICER was estimated to be €5,725, an essentially similar finding.

On the incremental cost-effectiveness plane (Figure 1), each data point represents one simulated ICER. Eighty percent of the simulated ICERs fall in the northeast quadrant, indicating that there is a 80% chance that GPs who receive the tailored intervention will recognise patients with anxiety or depression more often, but at additional cost. Twelve percent of simulated ICERs fall in the southeast quadrant, indicating a 12% chance that GPs who received the one-day training session and feedback will recognise patients with anxiety or depression more often, with less additional cost. The remainder of the simulated ICERs fall on the western side of the plane, indicating reduced effectiveness and lower costs (1%), or reduced effectiveness and higher costs (7%).

The difference between the control group and intervention group at baseline was caused by a few respondents with extreme values for monthly total cost (n=5: n=4 in the intervention condition and n=1 in the control condition). When these outliers were dropped from the analysis the mean total cost per participant per month was €702.14 in the control group and €840.26 in the intervention group; *p*=0.207. The mean ICER was estimated to be €5,001=€542/0.11 (after rounding) per additional recognised patient. Using the 2500 bootstraps, the median ICER was €4,492, an essentially similar finding to the primary analysis. A sensitivity analysis, encompassing the highest total cost per participant over a 6-month time, the lowest mean total cost per participant over a 6-month time frame, and the exclusion of patients’ from 6 GPs who did not receive the tailored intervention did not affect the overall conclusion that GPs who receive the intervention are more likely to recognise patients with anxiety or depression; but this incurs additional costs (Table 3).

**Statement of principal findings**

Cost effectiveness analysis showed that the incremental cost was €6,807 for each additional recognised patient in the intervention group. Sensitivity analyses confirmed the robustness of these findings.

Regarding the economic evaluation our study illustrates the importance of considering cost-effectiveness: tailoring requires additional resources, which should be offset by additional effects. Because of the relatively short duration of the data collection phase (6 months), it is unknown how the cost-effectiveness of the tailored intervention changed over longer periods. Tailored interventions may be a solution to improve the uptake of guideline recommendations for anxiety and depression in general practice but more research is needed before large scale tailored implementation can be recommended. Also the cost-effectiveness of the tailored intervention should be studied.

**Table 1. Direct medical and direct non-medical costs by health service type**

| Health service type | Direct medical costs | | Direct non-medical costs | | |
| --- | --- | --- | --- | --- | --- |
|  | Unit | Unit cost price^a^ (€) | Distance (km)^b^ | Time (h)^b^ | Unit cost price^c^ (€) |
|  |  |  |  |  |  |
| General practitioner | Contact | 28.35 | 1.1 | 1 | 15.69 |
| Company doctor^d^ | Contact | 28.35 | 17.6 | 0.5 | 9.89 |
| POH-GGZ General practitioner | Contact | 57.72 | 5 | 2 | 29.36 |
| Social worker | Contact | 65.80 | 5 | 2 | 29.36 |
| Private practice psychotherapist, psychiatrist | Contact | 91.14^e^ | 7 | 2 | 29.77 |
| Alcohol and drug consultant (CAD) | Contact | 173.12 | 10^f^ | 3 | 42.73 |
| Regional mental health service | Contact | 173.12 | 5 | 3 | 41.72 |
| Physiotherapist | Contact | 36.45 | 2.2 | 2 | 28.80 |
| Mental hospital | Contact | 175.19 | 7 | 3 | 42.43 |
| Medical specialist general hospital | Contact | 64.79 | 7 | 3 | 42.43 |
| Medical specialist academic hospital | Contact | 130.59 | 7 | 3 | 42.43 |
| Alternative treatment^g^ | Contact | 51.44 | 5 | 1 | 16.70 |
| Social Psychiatric Nurse | Contact | 81.01 | 1.1 | 1 | 15.91 |
| Home care | Hour | 35.44 | NA | NA | NA |
| Informal care (family, friends)^h^ | Hour | 12.66 | NA | NA | NA |

^a^ Integral unit cost prices [Hakkart-van Roijen et al (2010)] presented in 2010 € (with 1€ 2009 = 1.0127€ 2010).

^b^ Based on average distances (in special tariff taxi and public transport zones) and travel + waiting + treatment times (in hours) for receiving treatment [Hakkart-van Roijen et al (2010)].

^c^ Costs =  (0.2 × km) + 3 + (12.66 × hrs). With €0.20 = cost per km; €3 = 1 h parking time; €12.66 = 1 h time [Hakkart-van Roijen et al (2010)] presented in 2010 €.

^d^ No parking costs assumed.

^e^ Own calculation, valued as average of private practice psychotherapist and psychiatrist [Hakkart-van Roijen et al (2010)].

^f^ Assumed as CAD were more dispersed than regional mental health services.

^g^ Own calculation, valued as average of homoeopath and acupuncturist [Hakkart-van Roijen et al (2010)].

^h^ Valued as domestic help [Hakkart-van Roijen et al (2010)].

**Table 2 Cost distribution by groups at baseline and 6-month follow-up**

| Group |  | Test time costs^1^ | |
| --- | --- | --- | --- |
|  |  | Baseline, € (SD) (95%CI) | 6-month follow-up, € (SD) (95%CI) |
|  |  |  |  |
| Control group (n=246) | |  |  |
|  | Direct medical costs (incl. medication costs) | 270.34 (431.70) (216.13-324.56) | 192.16 (286.56) (156.17-228.14) |
|  | Direct non-medical costs | 112.08 (131.95) (95.51-128.65) | 76.12 (111.87) (62.07-90.17) |
|  | Productivity losses^2^ | 348.43 (975.66) (225.90-470.96) | 237.99 (905.51) (124.27-351.70) |
|  | Intervention costs | NA^4^ | 215.22 |
|  | Total costs^3^ | 730.85 (1147.07) (586.80-874.90) | 721.49 (1057.87) (588.63-854.33) |
| Intervention group (n=198) | |  |  |
|  | Direct medical costs (incl. medication costs) | 282.88 (311.05) (239.29-326.48) | 248.93 (334.66) (202.03-295.83) |
|  | Direct non-medical costs | 115.56 (124.33) (98.13-132.98) | 93.11 (118.80) (76.47-109.77) |
|  | Productivity losses^2^ | 588.64 (1459.73) (384.06-793.22) | 312.20 (894.49) (186.84-437.56) |
|  | Intervention costs | NA^4^ | 500.72 |
|  | Total costs^3^ | 987.08 (1596.15) (763.38-1210.78) | 1154.96 (1076.10) (1004.15-1305.78) |

^1^Mean costs on monthly basis presented in 2010 values; ^2^Presenteeism, absenteeism, all relate to productivity losses; ^3^Total costs are the sum of the other cost components. Differences in the totals are due to rounding; ^4^NA: not applicable

**Table 3 Sensitivity analysis of the incremental cost-effectiveness for different scenarios over a 6-month time frame**

|  | | Tailored implementation programme € (95%CI) | Alternative Scenarios^1^  € (95%CI) | | | | | | |
| --- | --- | --- | --- | --- | --- | --- | --- | --- | --- |
|  |  |  | A | | B | | C | | D |
|  |  |  |  | |  | |  | |  |
| Incremental cost-effectiveness ratio (ICER)^2^ |  | 6,807 (-273,002-336,608) | 5,001 (-293,358-361,275) | | 7,732 (-305,208-384,448) | | 6,369 (-297,616-370,617) | | 6,681 (-314,555-395,198) |
| Incremental Effectiveness |  | 0.11 | 0.11 | | 0.11 | | 0.11 | | 0.10 |
| Incremental cost-effectiveness ratio (ICER), median €^3^ | | 5,725 | 4,492 | | 6,739 | | 5,596 | | 5,560 |
|  | |  |  | |  | |  | |  |
| **Distribution on the cost-effectiveness plane (percent of the simulated ICER)** | | | | | | | | | |
|  | | Tailored implementation programme | Alternative Scenarios^1^ | | | | | | |
|  |  |  | A | B | | C | | D | |
|  | 1^st^ quadrant (northeast) | 0.80 | 0.77 | | 0.84 | | 0.80 | | 0.80 |
|  | 2^nd^ quadrant (inferior: northwest) | 0.07 | 0.06 | | 0.06 | | 0.07 | | 0.07 |
|  | 3^rd^ quadrant (southwest) | 0.01 | 0.01 | | 0.01 | | 0.01 | | 0.01 |
|  | 4^th^ quadrant (dominant: southeast) | 0.12 | 0.16 | | 0.09 | | 0.12 | | 0.13 |

^1^Intervention costs included in the estimation are respectively: (A) excluding outliers (B) the highest average per patient cost over a 6-month time frame; (C) the lowest average per patient cost over a 6-month time frame; (D) without the inclusion of patients’ from the 6 GPs in the intervention group who did not receive the tailored intervention. ^2^Mean cost for an additional recognition at 2010 prices. ^3^Median is 50th percentile of 2500 bootstrap replications of the ICER.

**Figure 1 Distribution of bootstrapped incremental cost effectiveness ratios (ICERs) (n=2500) on the cost effectiveness plane, primary analysis**

**References**

1. Hakkaart-van Roijen L: *Manual Trimbos/iMTA questionnaire for costs associated with psychiatric illness [in Dutch].* Rotterdam: Institute for Medical Technology Assessment; 2002.

2. Hakkaart-van Roijen L, Tan S, Bouwmans C: *Manual for cost research. Methods and standard cost prizes for economic evaluations in health care [in Dutch].* Diemen: College voor zorgverzekeringen; 2010.

3. College voor Zorgverzekeringen: *Pharmacotherapeutic Compass [in Dutch].* Diemen: College voor Zorgverzekeringen; 2009.
